# Additive Manufacturing Titanium Dental Implants Placed in Sinuses Grafted with 70HA:30-TCP: A One-Year Retrospective Study for Evaluation of Survival Rate

**DOI:** 10.3390/dj12060181

**Published:** 2024-06-13

**Authors:** Ilton José Mafra, Dimorvan Bordin, Rafael S. Siroma, Vittorio Moraschini, Leonardo P. Faverani, João Gabriel Souza, Carlos Fernando Mourão, Jamil Awad Shibli

**Affiliations:** 1 Department of Periodontology, Dental Research Division, Guarulhos University, Guarulhos 07023-070, SP, Brazil; intonmafra@gmail.com (I.J.M.); dimorvan_bordin@hotmail.com (D.B.); rafaelshinoske@gmail.com (R.S.S.); jgabriel.ssouza@yahoo.com.br (J.G.S.); 2 Department of Periodontology, Dental Research Division, School of Dentistry, Veiga de Almeida University, Rua Ibituruna, 108, Maracanã, Rio de Janeiro 20271-020, RJ, Brazil; vitt.mf@gmail.com; 3 Department of Diagnosis and Surgery, Division of Oral and Maxillofacial Surgery and Implantology, School of Dentistry, São Paulo State University (UNESP), R. José Bonifácio, 1193—V. Mendonca, Araçatuba 16015-050, SP, Brazil; leonardo.faverani@unesp.br; 4 Department of Periodontology, Tufts University School of Dental Medicine, Boston, MA 02111, USA

**Keywords:** maxillary sinus augmentation, synthetic material, dental implants, bone substitutes

## Abstract

The present short-term retrospective study evaluated the implant survival rate and peri-implant bone loss around additive-manufactured titanium implants placed in sinuses grafted with Plenum Osshp (Plenum Bioengenharia, Jundia, SP, Brazil) (70HA:30β-TCP) material. A total of 39 implants were inserted after 23 sinus floor elevation procedures in 16 consecutive patients. Prosthetic rehabilitation included fixed partial prostheses (three units), single crowns (eleven units), and fixed full arches (three units). Clinical and radiographic parameters of implant-supported restorations were evaluated after at least one year of occlusal loading. The implant–crown success criteria included the absence of pain, suppuration, and clinical mobility, an average distance between the implant shoulder and the first visible bone contact (DIB) < 1.0 mm from the initial surgery, and the absence of prosthetic complications at the implant–abutment interface. The overall cumulative implant survival rate was 97.43%. No prosthetic complications at the implant–abutment interface were reported. After one year, the mean DIB was 0.23 mm ± 0.14. Within the limits of this retrospective study, it can be concluded that 70 HA:30 β-TCP allowed stable and reliable bone support to maintain healthy conditions around titanium dental implants produced by additive manufacturing.

## 1. Introduction

The management of edentulous areas in the posterior maxilla generally provides limited bone height because of severe post-extraction alveolar crest resorption and age-linked sinus pneumatization [1,2]. These anatomic limitations may affect successful osseointegration and the fabrication of a functional and aesthetic implant-supported prosthesis, dictating the need for reconstructive osseous surgery to re-establish adequate bone volume for implant positioning [2]. To overcome these problems, various approaches, such as tilted implants, short implants, or grafting of the maxillary sinus floor, have been described in the literature [3,4,5].

Currently, grafting the maxillary sinus floor has become a reliable, commonly used surgical procedure to increase bone volume in the posterior maxilla [1,2,3], as it allows the placement of dental implants in sites that were previously considered unsuitable for implant insertion because of insufficient bone volume [2]. Several surgical techniques have been presented to assess the sinus cavity, elevate the sinus membrane, and place bone grafts [1,2,3]. In the lateral window sinus lift approach [6], a window is created through the buccal plate of the maxilla; the sinus membrane is carefully lifted, and autogenous bone (or bone substitute) is placed into the built space.

Although many bone graft materials have been used to fill the sinus cavity [7,8], essential questions about the possible presence of prions in xenograft materials, specifically from bovine origin, raise important issues [9]. Prion is a protein that can induce aberrant folding of other proteins in the brain. Prion-related diseases have the potential to impact both humans and animals, and there are instances where these diseases can be transmitted to humans through consuming contaminated products such as meat and organic graft material (xenografts). In a recent letter to the editor, Zenobio et al. [9] stated that the subjects who received xenograft material must be aware of the presence of these proteins and the possible risks for degenerative diseases. In addition, ethnic and cultural situations may require non-organic materials such as ceramics and polymers.

In this sense, the biomaterial field has made recent advancements and improved production methods for processing synthetic (bio)ceramics [10,11,12,13,14]. Recent advancements in biomaterial technology have particularly enhanced the production of synthetic (bio)ceramics, such as biphasic calcium phosphate materials combining hydroxyapatite (HA) and β-tricalcium phosphate (β-TCP), which offer optimized biological and mechanical properties suitable for bone replacement [7,12,13].

Hydroxyapatite, which shares similarities with bone tissue, is commonly used as a grafting material in bone regeneration because of its exceptional biocompatibility and osteoconductive properties [12,13]. β-TCP is desirable for bone regeneration in regenerative medicine due to its outstanding osteoconductive and osteotransductive capabilities. It possesses a crystalline structure that allows for easy breakdown and maintains thermal stability [14,15,16]. HA’s slow resorption rate hinders the bone neoformation process. Nevertheless, it can sustain its arrangement and framework for prolonged durations [16]. Conversely, β-TCP has a high degree of solubility and quick resorption, which promotes bone production but hampers the preservation of the framework [17,18].

Combining HA and β-TCP can produce biphasic calcium phosphate ceramics with different phase compositions (HA:β-TCP ratios). These ceramics, known as CaP ceramics, are highly significant in dentistry and medical fields due to their biocompatibility and osteoconductivity [14,17,18]. The characteristics of ceramics can be altered by adjusting the HA:β-TCP ratio and other factors such as the Ca:P ratio, structure, surface, porosity, and chemistry. This allows for the creation of materials with unique properties, including customizable resorption activity, mechanical strength, porosity, and granulometry [18,19]. Complementary to these, an essential requirement for an optimal graft material is an extensively interconnected porous structure, ranging from 50 to 400 microns, with sufficiently wide pore sizes and linkages, allowing cell migration, fluid exchange, tissue ingrowth, and vascularization with the penetration of blood vessels [17,19].

In addition, recent studies have shown that titanium dental implants produced by additive manufacturing presented superior bone-to-implant contact, especially in type IV bone [20]. The implant surface topography is made by a high-power laser, melting the titanium particles, and resulting in a unique surface that provides better bone tissue anchorage [20,21,22,23].

Therefore, this retrospective study evaluated the survival rate, peri-implant bone loss, and prosthetic complications, after a one-year follow-up, of additive manufacturing titanium dental implants placed in grafted sinuses with biphasic calcium phosphate ceramics in a 70HA:30 β-TCP ratio as the only bone graft material.

## 2. Materials and Methods

### 2.1. Patient Population

The present study evaluated the impact of the biphasic synthetic bone graft material with a 70HA:30β-TCP ratio after maxillary sinus augmentation on the dental implant survival rate and peri-implant bone loss after one year of occlusal loading. Due to the nature of the study design (single-center and retrospective study), the sample size was determined by the availability of suitable cases that met the inclusion criteria within the clinical setting. Despite the limited sample size, all the procedures conformed rigorously to the ethical standards delineated in the Declaration of Helsinki for research involving human subjects. All the patients received a thorough explanation and signed a written consent form before enrolment in the study. This retrospective study was approved by the ethics committee of Guarulhos University (CEP-UnG process # 66307822.6.0000.5506).

Between December 2019 and October 2021, the patients referred to the Clinical Centre of Guarulhos University, Guarulhos, SP, Brazil, for maxillary sinus augmentation due to a lack of alveolar bone for the placement of dental implants were considered for inclusion in this retrospective clinical study. As inclusion criteria, only patients ≥21 years old, of both genders, in good systemic health, and with residual bone height between the sinus floor and alveolar ridge lower than 3 mm were considered. Exclusion criteria consisted of poor oral hygiene, active periodontal infections, active sinus infection, a history of persistent sinus infections, uncontrolled diabetes, and bruxism.

In this retrospective study, a total of 39 titanium dental implants were evaluated after being placed in 16 patients who underwent 23 sinus augmentation surgeries. These details provide a foundation for understanding the scope and scale of the research conducted.

### 2.2. Pre-Operative Procedures and Maxillary Sinus Augmentation

Dental surgeons carried out a comprehensive examination of the hard and soft tissues inside the oral cavity of each patient. Cone beam computed tomography (CBCT) scans generated by the KODAK 9500 Cone Beam 3D System (Carestream Dental LLC, Atlanta, GA, USA) were utilized to carefully evaluate the bone volume, bone quality, anatomy, and any existing sinus pathology or abnormalities. Before the procedural intervention of sinus augmentation, the patient thoroughly rinsed their oral cavity with 0.12% chlorhexidine gluconate antimicrobial oral rinse (PerioGard, Colgate, SP, Brazil) for one minute. All the patients mandatorily received a postoperative regimen of antibiotic therapy after the surgical sinus augmentation procedure: 1 g of amoxicillin trihydrate and potassium clavulanate tablets (Clavulin BD 875 mg, GlaxoSmithKline plc, Rio de Janeiro, RJ, Brazil) BID for seven days [19]. All the patients were treated under effective local anesthesia, using Mepivacaine 2% with epinephrine 1:100,000 (DFL, Rio de Janeiro, RJ, Brazil) administered in the region undergoing the procedure. A trapezoidal incision outline was made, followed by gentle periosteal elevation and mucosal detachment with a periosteal elevator molt surgical instrument (Polachini Instruments, Sao Paulo, SP, Brazil) to adequately expose the alveolar bone crest and allow an optimal view of the maxillary sinus’ crest and anterior bony wall. The access window was meticulously created via piezosurgery corticotomy using the cutting-edge Piezosurgery medical device (Piezosurgery Touch, Acteon Group, Merignac, France). Afterward, the integrity of the sinus membrane was maintained as it was gently detached from the floor of the maxillary sinus, carefully using proper sinus curette instruments (Polachini Instruments, Sao Paulo, SP, Brazil) and piezosurgery tips (Acteon Group, Merignac, France) to prevent any inadvertent damage. Extreme caution was exercised to strictly avoid perforation of the highly delicate Schneiderian membrane tissue layer. In the unfortunate incident of an intraoperative sinus membrane perforation, a bioresorbable synthetic polydioxanone (PDO)-based polymeric membrane (Plenum Guide, Plenum Bioengenharia, Jundiaí, SP, Brazil) was immediately utilized to seal the perforation and securely contain the bone graft material. The mucosa tissue layer lining the sinus floor was gently separated from the underlying bony surface using a curved elevator instrument (Polachini Instruments, Sao Paulo, SP, Brazil). In all the surgical cases undergoing maxillary sinus augmentation, after the completion of the elevation of the sinus floor, the newly created enlarged chamber between the alveolar bone of the maxillary process and the relocated sinus floor was packed completely and filled to 100 percent capacity with moderate compression and compaction of the biphasic osteoconductive bone graft material, which comprised a 70 hydroxyapatite to 30 beta-tricalcium phosphate ratio (Plenum Osshp, Plenum Bioengenharia, Jundiai, SP, Brazil), to minimize residual dead spaces inside the modified sinus cavity. Subsequently, the lateral bony window access site was sealed using a bioresorbable PDO-based membrane (Plenum Bioengenharia, Jundiai, SP, Brazil), and the full-thickness mucoperiosteal flaps were precisely realigned and sutured using 4-0 nylon sutures (Microsuture, Sao Paulo, SP, Brazil) [19]. The sutures were removed after 10 days during a routine follow-up examination.

### 2.3. Dental Implant Design and Placement

**Figure 1:**
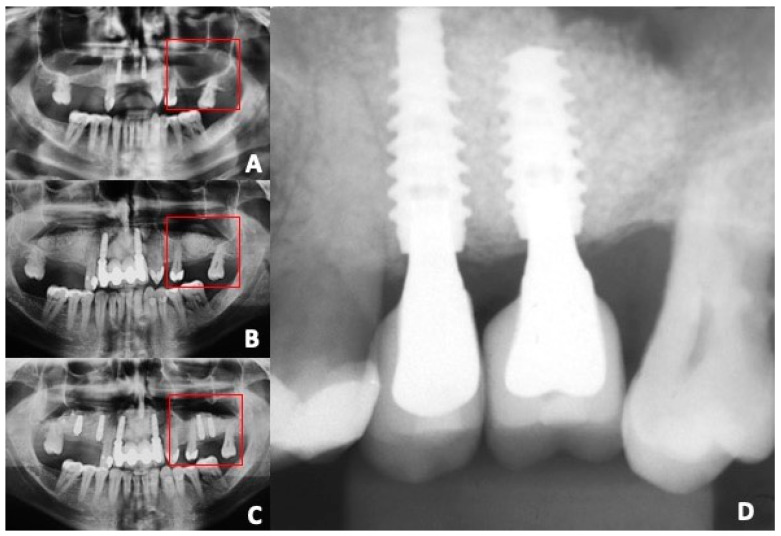
Radiographic view of the different stages of the maxillary sinus augmentation: (**A**) extensive pneumatization of the left side of the posterior maxilla; (**B**) 6 months after maxillary sinus augmentation with biphasic material showing a sufficient amount of newly formed bone; (**C**) 3 months after implant placement in the second premolar and first molar in newly formed bone after bone regeneration; (**D**) intra-oral radiographic image after 1 y loading showing no bone loss resorption around the implant–abutment connection. Red squares depicted the maxillary sinus augmented area.

This study included dental implants manufactured from titanium alloy (Ti6Al4V ELI) powder produced by the additive manufacturing technology of laser melting (Plenum Bioengenharia, Jundiai, SP, Brazil). The insertion of the dental implants occurred afterward, allowing a healing period of 5 to 6 months after performing the maxillary sinus augmentation procedures. The dental implant’s diameter and length were determined based on an evaluation of new bone formation using a CBCT scan obtained before the implant placement. Preparation of the implant sites in the maxillary bone was undertaken with spiral drills of incrementally increasing diameter, beginning with 2.0 mm drills and progressing up to 2.8 mm drills; then, conical osteotomes (Plenum Bioengenharia, Jundiai, SP, Brazil) were utilized to widen the sites to ultimately place the dental implants, which measured 3.5 mm, 4.0 mm, and 4.5 mm in diameter, under constant irrigation with sterile saline solution. The dental implants produced by Plenum Bioengenharia were then carefully positioned at an infra-crestal level of 1 to 3 mm below the maxillary bone crest (Figure 1). The mucoperiosteal flaps were precisely repositioned and sutured utilizing non-resorbable sutures (polytetrafluoroethylene (PTFE) 4-0) Microsuture, Sao Paulo, SP, Brazil), thereby submerging and completely covering the implants. In all the clinical cases, the postoperative pain was controlled by administration of 200 mg of nimesulide anti-inflammatory medication (Aché, Rio de Janeiro, RJ, Brazil) every 12 h for 4 days, and detailed oral hygiene instructions were provided to the patients, including a recommendation for mouth rinses with 0.12% chlorhexidine oral rinse solution (PerioGard, Colgate, São Paulo, SP, Brazil) two times a day for seven days. The sutures were removed at 10 days post-surgery without incident.

### 2.4. Implant-Supported Restorations

Following a 3-month healing period after implant placement, the dental implants were transferred for prosthetic abutment selection according to each case (Plenum Bioengenharia, Jundiaí, SP, Brazil). Full-arch polyether impressions (Impregum, 3M ESPE, St. Paul, MN, USA) were made using custom open-tray impression components and, if necessary, splinted together (in the case of fixed partial prostheses and fixed full arches) with autopolymerizing acrylic resin (Triad TruTray, DENTSPLY, York, PA, USA). Working casts poured in ADA Type IV dental stone (ResinRock, WhipMix, Louisville, KY, USA) were utilized by the laboratory team to fabricate screw-retained provisional restorations using autopolymerizing polymethyl methacrylate acrylic resin (Alike, GC America, Alsip, IL, USA). The occlusal adjustment was meticulously performed using a combination of clinical examination and digital occlusal analysis to identify and eliminate premature contacts during maximum intercuspation and lateral and protrusive movements. This precision ensures the minimization of off-axis loading, which is critical to prevent mechanical complications and optimize load distribution during the initial healing period and subsequent transition to definitive prostheses. Master casts and bite registrations were obtained using a fast-setting vinyl polysiloxane bite registration material (Futar D, Kettenbach GmbH, Eschenburg, Germany). Definitive implant-supported single crowns were milled from monolithic translucent zirconia blanks (Katana Zirconia ML, Kuraray Noritake, Tokyo, Japan) and stained to match the natural dentition characteristics of each patient. The final implant-supported restorations were cemented over titanium alloy abutments (Plenum Bioengenharia, Jundiaí, Brazil) that had been torqued to 20Ncm using composite resin cement (RelyX Unicem 2, 3M ESPE, St. Paul, MN, USA) per the manufacturer’s guidelines. Before discharging the patient, the occlusal contacts were thoroughly examined using articulating paper and verified with intraoral digital scanning. This verification process guarantees that the occlusal forces are balanced, which helps to reduce the likelihood of undue stress on the implant–bone interface. Such stress is a known risk factor in peri-implant bone loss and implant failure. Patients were placed on a clinical recall schedule to monitor the function of the implant restorations.

### 2.5. Clinical and Radiographic Evaluation

After one year of loading, the implants were evaluated using the dichotomous clinical parameters: presence or absence of pain, sensitivity, suppuration, and implant mobility.

Moreover, intraoral periapical radiographs were taken for each implant, using a Rinn alignment system with a rigid film–object X-ray source coupled to a beam-aiming device to achieve reproducible exposure geometry. Radiographs were taken at the baseline (day of final implant-support restoration installation) and one-year follow-up session for two purposes: to evaluate the presence/absence of continuous peri-implant radiolucency; to measure a mean of the distance between the implant shoulder and the first visible bone contact (DIB) in mm at the mesial and distal implant site [24]. The images were measured using Version 2.1 ImageJ software (Wayne Rasband, National Institute of Mental Health, USA). For the second measurement, crestal bone level changes were recorded as changes in the vertical dimension of the bone around the implant so that an evaluation of peri-implant crestal bone stability was achieved with time. To correct for dimensional distortion in the radiograph, the apparent dimension of each implant (directly measured on the radiograph image) was compared with the actual implant length and the following equation [24]:

Rx implant length: True implant length = Rx DIB: True DIB

## 3. Results

### 3.1. Surgical Interventions and Patient Population

The 39 dental implants were placed in the healed maxillary sinuses following lateral wall sinus augmentation using Plenum Oss (Plenum Bioengenharia, Jundia, SP, Brazil) biphasic bone graft material. At baseline prior to the sinus lifting, the mean residual bone height was 1.98 ± 1.03 mm. The staged implant placement procedure allowed for progressive bone regeneration and maturation over the 5–6 month-healing period before surgical re-entry. During implant site preparation and fixture insertion, abundant regenerated vital bone was observed filling the subantral space, clinically confirming the biocompatibility and osteoconductivity of the 70HA:30β-TCP synthetic graft. The mean final vertical bone height prior to implantation was 13.4 ± 3.7 mm, reflecting excellent volume stability maintenance and substantial de novo bone growth induced by the biphasic ceramics.

In total, sixteen subjects (four men and twelve women, aged between 34 and 61 years, average 48.3 ± 13.3 years) were enrolled. A total of 23 sinus augmentation procedures were performed. Three sinuses presented small sinus membrane perforations, and they were treated with interposition of bioresorbable PDO-based membrane (Plenum Guide, Jundiai, SP, Brazil) before graft insertion and did not present infection during the healing period. The distribution of implants by localization, length, and diameter is shown in Table 1 and Table 2. The various indications for implant therapy are listed in Table 3. Each fixed full-arch prosthesis was supported by 6 to 8 implants, some of them out of the grafted sinuses. The fixed partial and full-arch prostheses were ceramic–metallic; the single crowns were ceramic–metallic or monolithic ceramic.

### 3.2. Implant Survival Rate and Peri-Implant Bone Loss

Among the 39 dental implants, most were 10 or 11 mm long, with only 8, 13, and 15 mm fixtures accounting for the remainder. The majority had 3.5 or 4.0 mm diameters, with only one 4.5 and one 5.0 mm wide implant placed. This size distribution highlights the capability of mature 70HA:30β-TCP-augmented maxillary bone to accommodate standard diameter solid screw implants 10–15 mm long, whereas longer or wider options may be limited. The 38 successfully osseointegrated fixtures exhibited no clinically detectable mobility, suppuration, or peri-implant radiolucency after one year of loading. The overall 97.4% cumulative survival rate and excellent soft and hard tissue responses support the suitability of additive manufacturing titanium implants in regenerated Plenum Oss bone graft material. The failed implant was removed at the second stage of surgery. The remaining implants did not cause pain or exhibit clinical mobility, suppuration, or exudation, with a DIB < 1 mm, and did not have any prosthetic complications at the implant-abutment interfaces. No prosthetic abutments became loose during this study. The peri-implant bone loss after one year of loading was 0.23 ± 0.14 mm.

## 4. Discussion

This study evaluated the success of 3D-printed titanium dental implants placed in sinuses grafted with biphasic calcium phosphate ceramics. The study assessed the implants’ survival rate, peri-implant bone loss, and prosthetic complications over a one-year period. The study demonstrated that biphasic bone graft material can support and maintain the implant-supported restoration after one year of loading. Although maxillary sinus augmentation is a well-documented procedure [7,8], this is the first study to describe the short-term evaluation of this synthetic biphasic bone graft material in human maxillary sinuses. In addition, to the best of our knowledge, this study is the first to investigate additive manufacturing titanium implants following maxillary sinus lifting and graft material filling under the lowest bone density conditions for oral rehabilitation.

The clinical outcomes show a higher survival rate (97.43%) and a peri-implant bone loss of 0.23 ± 0.14 mm, which is in agreement with previous data that used different graft materials [7,8,25]. Two other studies evaluated the use of autogenous bone mixed with bioceramics, xenografts, or autologous platelet concentrates for sinus lifting procedures, with a follow-up of at least one year after implant loading. The implant survival rates in these studies ranged between 90% and 96.5% [26,27].

Several graft materials have been used during maxillary sinus augmentation, with a high success rate in bone gain after long-term periods [8,10]. The majority of the studies evaluated xenograft materials with or without autogenous bone [28,29], showing higher clinical outcomes. However, the desires and expectations of patients are crucial in the field of reconstructive and regenerative surgeries. Given the potential for disease transmission when using xenograft material and the fact that patients may reject them for several reasons, such as vegetarianism, veganism, and religious concerns, it would be preferable to have a synthetic graft material as an alternative that offers predictable and comparable results [9,28].

Regarding the potential risks for disease transmission using xenografts, despite these materials undergoing extensive processing to eliminate cellular components, such as decellularization and sterilization, the complete elimination of all potential risks is challenging. While the literature often portrays it as a low-risk factor, it is important to note that the risk is not entirely absent [30,31]. Proteins were detected in certain xenograft materials, including TutoPlast, Bio-Oss, and tibia samples processed using a similar deproteinization method as employed in Bio-Oss. Furthermore, the complete proof of bovine spongiform encephalopathy prion inactivation has not been established. Some authors propose discontinuing the use of bovine bone based on this uncertainty [30,31]. As discussed by Zenóbio et al. [9], the use of an engineered ceramic graft offers several advantages over traditional bone grafting techniques. This fully synthetic bone substitute eliminates issues with religious objections, risk of prion disease transmission from bovine sources, and comorbidity from autograft harvest sites, providing a biocompatible and ethical solution that meets the expectations of modern patients.

Previous studies in humans [32,33], including a human autopsy [33], showed the histological and clinical behavior of biphasic material was effective, showing lamellar bone surrounding the remaining HA particles, suggesting that it served as scaffolding, and preserving its osteoconductive capabilities until it degraded. This process resulted in a stable graft material after 5 to 6 months of healing and allowed the dental implant placement. In addition, the maxillary graft augmentation using Plenum Oss resulted in a vertical bone gain that allowed the placement of an implant of 13 to 15mm in length (Table 2), increasing the dental implant stability.

Complementary, additive-manufactured implants placed in augmented maxillary sinus with Plenum Oss presented a very stable peri-implant bone margin after one year of occlusal loading. This result was also potentialized by the unique characteristic of the implant surface topography, which enhances the bone-to-implant (BIC) contact, as previously described [20,21,22,23]. Human histology of a 3D-printed titanium implant surface showed an increased bone-to-implant contact percentage not only due to its higher roughness but also to the interconnected titanium particles (TiGr23) that presented bone ingrowth [21,22]. Despite the existence of several well-established dental implants, ongoing efforts are needed to enhance their microstructure for better outcomes, particularly in regions with low bone density [34,35].

The biphasic ceramics in this study had an interconnected porous structure, an optimized calcium-to-phosphate ratio, and controlled solubility. These characteristics made them highly conducive to bone formation and allowed for cellular migration, angiogenesis, and bone ingrowth while maintaining volume stability over six months [13,33]. This enabled sufficient bone formation before the implant placement. Furthermore, the use of Plenum Oss synthetic biphasic graft bone material in maxillary sinus augmentation procedures resulted in significant vertical bone gains, averaging 10 to 15 mm. This allowed for the placement of dental implants to increase stability, even in areas with low residual bone height before surgery. The graft material likely maintained sufficient structural integrity over the 5–6 months of healing before implantation, facilitating bone deposition through its interconnected macroporosity. Additive-manufactured implants have the potential to enhance mechanical strength and corrosion resistance, which are crucial factors for success in the field [22]. However, the study has certain limitations, such as a modest cohort size and a single-center retrospective study. Consequently, the findings should be interpreted cautiously regarding generalizability. Moreover, the limited follow-up period necessitates confirmation of whether stable peri-implant bone levels and healthy soft tissue attachments are maintained long-term.

To enable more consistent, quantitative assessments of Plenum Oss performance, standardizing variables like residual alveolar height or absolute vertical bone gain can be helpful. Ideally, stratification by age, sex, smoking status, and other demographic factors can also be performed. Additionally, comparing additive manufacturing implants to machined surfaces can help isolate the specific impact of 3D laser microtexturing.

Overall, these positive initial findings support the use of synthetic biphasic ceramics and innovative titanium fixtures for adjuvant maxillary sinus reconstruction and rehabilitation.

## 5. Conclusions

- This retrospective study, at the one-year follow-up, found that 70% hydroxyapatite (HA) and 30% beta-tricalcium phosphate (β-TCP) provided stable bone support for titanium dental implants made using additive manufacturing. Plenum Oss, a synthetic biphasic bone graft material, allowed significant bone regeneration in the maxillary sinus, making it easier to place dental implants that achieved excellent osseointegration. The additive-manufactured grade-23 titanium implants had a survival rate of 97.43% after one year of loading in grafted areas.
- The additive manufacturing process for the titanium implants produced a complex topography and rough surface, which provided ideal bone anchorage, even in low-density bone. This contributed to the excellent clinical results, with an average distance to the first bone contact of only 0.23 mm, and no instances of implant failure or peri-implant infections among the remaining 38 successful fixtures.
- In summary, this study supports the efficacy of synthetic HA:β-TCP biphasic bone grafts and innovative additive manufacturing techniques in maxillary sinus augmentation and implant rehabilitation. Plenum Oss HA:β-TCP ceramics and additive-manufactured titanium implants achieved excellent clinical performance, making them a viable solution for grafts and implants. Synthetic alternatives like these address the modern expectations of patients while enabling predictable restorative therapy, even in anatomically challenging cases requiring maxillary sinus reconstruction. However, further studies are necessary to validate these results, investigate long-term outcomes, and determine any demographic variances.

## Figures and Tables

**Table 1:** Localization of 39 inserted implants in the posterior maxilla.

Implant Sites	No. of Implants
First premolar	08
Second premolar	15
First molar	15
Second molar	01
Total	39

**Table 2:** Distribution of implants by length and diameter.

Diameter (mm)	Length (mm)				
	8	10	11	13	15
3.5	2	2	3	5	4
4.0	2	3	2	5	1
4.5	1	2	1	3	1
5.0	1	1	-	-	-

**Table 3:** Indication for the placement of 39 implants in the posterior maxilla.

Type of Restoration	No. of Units	No. of Implants
Single-tooth restorations	11	12
Fixed partial prostheses (FPPs, 2–4 elements)	03	17
Fixed full arches	03	10
Total	17	39

## Data Availability

This study did not generate supporting data.
